# Cosmetic outcomes of postoperative scar taping after surgical incisions: A systematic review

**DOI:** 10.1016/j.jpra.2026.07.002

**Published:** 2026-07-08

**Authors:** David Kwan Ryung Lee, Dave Osinachukwu Duru, Gaetan Nicolet, Cheuk Ying Kyleen Kiew, Ahid Abood, Kai Yuen Wong

**Affiliations:** a School of Clinical Medicine, University of Cambridge, Cambridge, United Kingdom; b University of Lausanne, Faculty of Biology and Medicine, Lausanne, Switzerland; c Imperial College London, South Kensington, Ayrton Road, London, United Kingdom; d Department of Plastic and Reconstructive Surgery, Addenbrookes Hospital, Hills Rd, Cambridge, United Kingdom

**Keywords:** Cicatrix, Scar management, Tape, Micropore, Steri-Strip, Cosmetic outcomes

## Abstract

**Background:**

The application of silicone sheets to surgical wounds is currently recommended for hypertrophic scar and keloid prophylaxis after closure of surgical incisions. The use of other types of tapes that differ in cost and biomechanical properties has been reported, although a formal synthesis on this topic within recent years has yet to be undertaken.

**Methods & materials:**

MEDLINE, Embase and Cochrane were comprehensively searched between January 2000 – December 2025, following the PRISMA guidelines. Included studies evaluated cosmetic outcomes of adult patients (≥18 years) undergoing surgical wound taping following primary wound closure. Randomised controlled trials, comparative cohort studies, and case series (n≥5) were eligible.

**Results:**

In the 14 included studies, microporous tapes and Steri-Strips were most popular, encompassing 217 and 159 patients respectively. Cosmetic outcomes were assessed at a mean of 5.13 months (range 21 days to 12 months), using heterogeneous tools including SCAR-Q, Patient and Observer Scar Assessment Scale (POSAS), Vancouver Scar Scale, and Visual Analogue Scales. The application of tapes after primary closure showed significant improvements in scar cosmesis compared to patients without any interventions (n=75), with some evidence suggesting that more inexpensive paper tapes produce clinically similar results to gold-standard silicone sheets.

**Conclusion:**

Surgical wound taping using adhesives other than silicone sheets appears to be an effective strategy for improving scar cosmesis. Further randomised controlled trials studies using a consistently layered primary closure methods and directly comparing different types of tapes including silicone sheets using longer follow up and standardised reporting outcomes are needed.

## Introduction

The appearance of scars has an important impact on the psychosocial wellbeing of patients.^1^ Although scarring is a natural, unavoidable consequence of surgical incisions, their appearance can vary. Adverse scars are hypertrophic or keloid in nature and present with physical symptoms of discomfort, including pruritus, pain and stiffness.^2^ Algorithms for managing adverse scar formation have been proposed, ranging from prevention to treatment including surgical excision, laser therapy, and corticosteroid incisions depending on scar aetiology and morphology.^3^, ^4^, ^5^ Preventative algorithms generally include the application of silicone gels, hypoallergenic microporous tape and/or onion extract.

Silicone sheets are widely recognised as the first-line tool in adverse scar management.^3^, ^4^, ^5^ However, this material presents with several disadvantages. For example, silicone sheets cannot be applied immediately after operation before re-epithelialization^6^ and typically seen as higher maintenance compared to tapes.^7^ Their mechanism of action is thought to arise from both the tension reduction and the maintenance of a moist-healing environment.^7^

Tapes are typically used as an adjunct to primary wound closure rather than a direct method of wound apposition itself.^8^ The latter usage is less reliable due to the loss of adhesiveness that can arise^9^^,^^10^ and is therefore typically reserved for low-tension, superficial wound closure. Tapes have been used for modulating scar cosmesis, with some evidence suggesting that they may be as clinically effective as silicone sheets, if not more.^11^, ^12^, ^13^

Tapes can vary in terms of the materials they are made of, leading to differences in tension, moisture permeability and adhesiveness, and are therefore expected to modulate scar cosmesis to varying degrees.^14^ To our knowledge, no publication has focused on specific tape categories and their individual abilities to modulate post-operative scar formation.

## Methods

### Study protocol

This systematic review was performed following the Preferred Reporting Items for Systematic Reviews and Meta-Analyses Extension (PRISMA) and prospectively registered at PROSPERO (CRD:420261351256).

### Systematic search

Embase, MEDLINE and Cochrane Central Register of Controlled Trials were searched from January 2000 – December 2025 using a strategy, developed with the help of a medical librarian, that included free-text words such as “tape”, “scar” and “cicatri*” – see Supplementary Figure A for the full search strategy, adapted to each database. Studies to be included were randomised controlled trials, retrospective or prospective cohort studies, case-control studies and case series with 5 or more patients. The reporting of cosmetic outcomes, either through validated objective measures such as SCAR-Q or scar width/height, as well as the application of tape to prevent scar formation, were necessary criteria for inclusion. Conference abstracts without full text access, as well as non-human studies and short communications were excluded.

### Data extraction

Screening and data extraction were performed independently by two reviewers (D.K.R.L. and D.O.D.), using Rayyan AI and Excel software (version 16.106.2) respectively. All conflicts were resolved through discussion or by asking a third author (C.Y.K.K.). Extracted data included study characteristics (name and sample size), wound closure techniques, type of tape applied, timings of tape application, incision location, surgical category, cosmetic outcome measured, timings of cosmetic evaluations, and the reported cosmetic outcomes themselves.

### Risk of bias assessment

Similarly, risk of bias was assessed independently by two reviewers (D.K.R.L and G.N.), with any discrepancies being resolved through discussion with a third author (D.O.D.). The Cochrane Risk of Bias 2.0 and ROBINS-I tools were used to assess for randomised and non-randomised studies respectively.

## Results

### Literature search

**Fig. 1:**
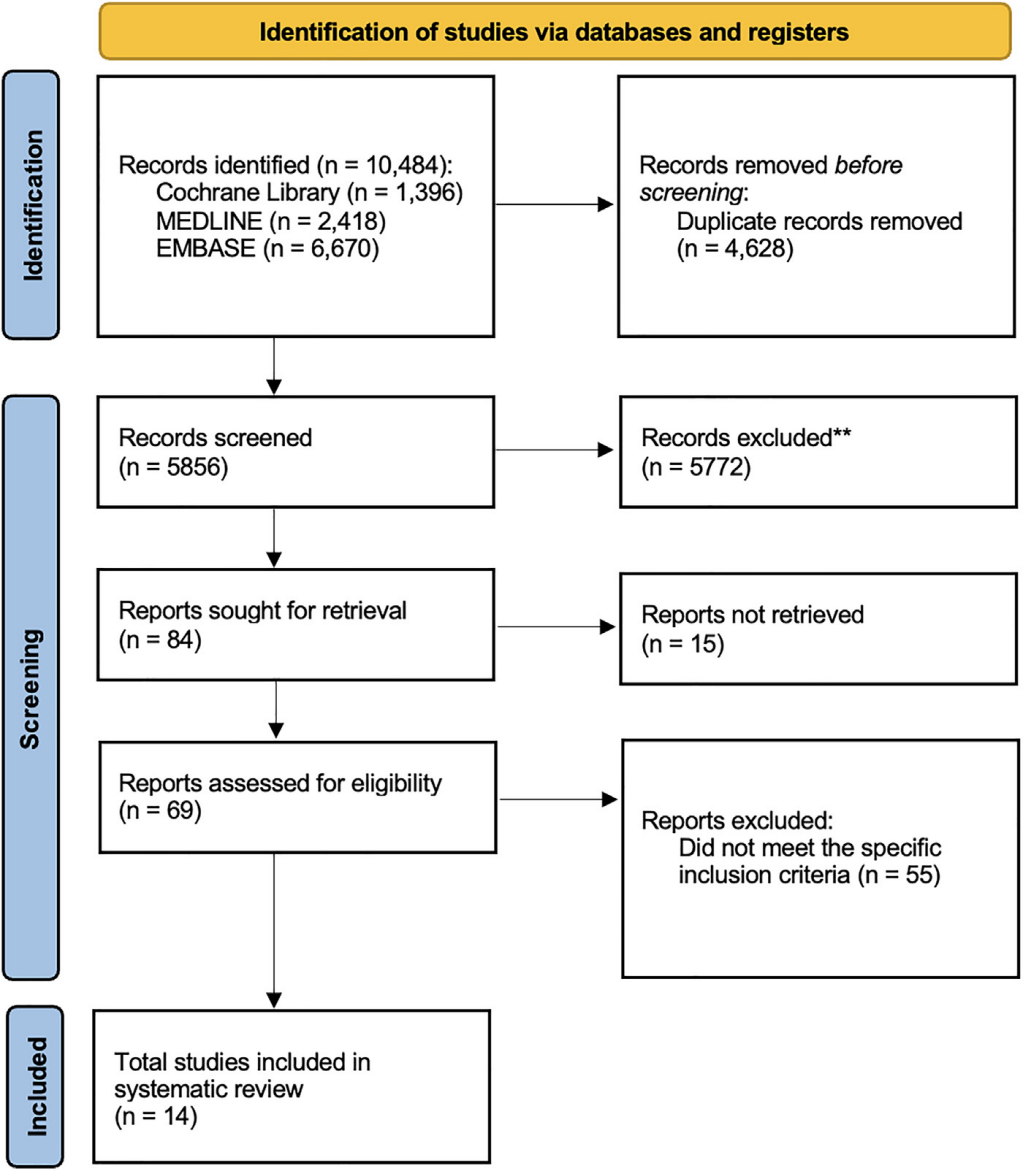
PRISMA flow diagram of the literature screening process.

A PRISMA flow diagram indicating the literature screening process is shown in Fig. 1. The search strategy yielded a total of 10,484 studies, of which 5,856 papers were screened after the removal of duplicates. 69 full-text papers were retrieved, of which 14 met all inclusion criteria and underwent data extraction. The dates of included studies ranged from 2001 to 2025, with the median year being 2022. Of note, the majority of included studies were level I randomised controlled trials (n=10) – see Table 1, Table 2, Table 3 for summaries of the included study characteristics.

**Table 1:** Summary of included studies for microporous tapes.

Authors and year	Year	Study design	Level of evidence	Total sample size	Surgery (location of tape)	Closure method for both control and tape groups	Intervention		Timing and duration of tape application	Cosmetic outcome	Timing of Outcome Measurement Posoperation
							Control group	Tape group			
Atkinson et al^16^	2005	Randomised controlled trial	I	14	Caesarean section (abdomen)	Not reported	No tape	Micropore tape^^^	After suture removal; 12 weeks	Scar volume	6 months
Chen et al^17^	2001	Prospective comparative cohort study	II	150	Elective colorectal surgery (abdomen)	•Deep fascia: 1-0 polydiosanone monofilament*	Interrupted skin sutures: silk (Unik, Milwaukee, Wis)	Micropore tape^^^	At wound closure; 7-10 days	Satisfaction with scar; Narrowest width; Widest width	6 months
Gallo et al^18^	2025	Randomised controlled trial	I	11	Deep inferior epigastric perforator flap breast reconstruction (abdomen)	•Deep fascia: 3-0 vicryl* •Deep Dermis: 4-0 monocryl* •Subcuticular: 5-0 monocryl*	Closed incision negative pressure therapy	Micropore tape^	At wound closure; 7 days	SCAR-Q	6 months
Ilori et al^15^	2022	Randomised controlled trial	I	32	Benign tumour excisions, Open reduction internal fixations, Corrective osteotomies, arthroplasties (limbs)	•Subcutaneous: 2-0 polyglactin 910 •Skin: Staples or 3-0 polypropylene	No tape	Micropore tape^^^	After suture removal; Not reported	Scar width, Scar height, Scar category	6 months
Widgerow et al^19^	2009	Randomised controlled trial	I	10	Breast augmentation, Breast reduction (breast)	Not reported	Scar gel + microporous tape	Microporous tape	At wound closure; 3-6 months	Patient and observer scar assessment; morphologic grading system	6 months

**Table 2:** Summary of included studies for Steri-Strips.

Authors and year	Year	Study design	Level of evidence	Total sample size	Surgery (location of tape)	Closure method for both control and tape groups	Intervention		Timing and duration of tape application	Cosmetic outcome	Timing of Outcome Measurement Postoperation
							Control group	Tape group			
Gevel et al^21^	2010	Randomised controlled trial	I	39	Coronary artery bypass graft (chest)	•Subcutaneous: running absorbable monofilament synthetic structure	No tape	Steri-Strip^^^	At wound closure; 2 weeks	Visual analogue scale	6 weeks
Lazar et al^20^	2011	Randomised controlled trial	I	18	Median sternotomy (chest)	•Subcutaneous: 3-0 vicryl* •Epidermis: 4-0 vicryl*	No tape	Steri-Strip^^^	At wound closure; 7-10 days	Cosmesis scale (0-4)	21 days
Lin et al^38^	2020	Randomised controlled trial	I	47	Caesarean section (abdomen)	Not reported	Silicone (CICA-CARE; Smith & Nephew, Fort Worth, Texas);	Steri-Strip^^^	Applied 1 week post-operatively; 3 months	Vancouver scar scale; Visual analogue scale	12 months
O’Leary et al^23^	2013	Randomised controlled trial	I	32	Thyroid/parathyroid tumour excision (neck)	•Subcutaneous: 2-0 vicryl*	Subcuticular suture (4–0 Monocryl; Ethicon, Inc)	Steri-Strip^^^	At wound closure; 1-2 weeks	Hollander cosmesis scale; Likert scale	6 weeks
Ramdhan et al^22^	2013	Randomised controlled trial	I	23	Open reduction internal fixation of femur fractures (lower limb)	•Deep fascia: 1-0 vicryl •Subcutaneous: 0-0 vicryl	Interrupted skin sutures: Dafilon 3-0	Steri-Strip^^^	At wound closure; 2 weeks	Mean scar width difference; Mean wound length difference	12 weeks

**Table 3:** Summary of included studies for other tapes.

Authors and year	Year	Study design	Level of evidence	Total sample size	Surgery (location of tape)	Closure method for both control and tape groups	Intervention		Timing and duration of tape application	Cosmetic outcome	Timing of Outcome Measurement Postoperation
							Control group	Tape group			
Ishii et al^24^	2021	Prospective comparative cohort study	II	163	Tissue expander insertion, Silicone breast implant insertion (breast)	Not reported	Hypoallergenic polyester-woven Atofine™ (Nichiban, Tokyo, Japan)	Nonwoven surgical tape Yu-ki ban (Nitto Medical, Tokyo, Japan)	After suture removal; 4 months	Scar width; Vancouver scar scale	5 months
Klingenstein et al^25^	2022	Randomised controlled trial	I	8	Dermis fat grafting (gluteal region)	•Subcutaneous: 2-0 vicryl (Ethical GmbH, Norderstedt, Germany) •Epidermis: 2-0 silk	Steri-strips	Kinesio tape (Atex medical)	Not reported; 4-6 weeks	Mean scar length; prominence; colour; mean observer grading	3 months
Shiva et al^27^	2023	Randomised controlled trial	II	65	Elective hernia surgery (abdomen)	Not reported	No tape	Tape not specified	Not reported; Not reported	Hollander wound evaluation; visual analogue score, patient satisfaction, surgeon satisfaction	3 months
Su et al^26^	2025	Retrospective comparative cohort study	III	29	Tattoo resection (forearm)	•Deep: 3-0 PDS-II •Shallow: 4-0 PDS-II •Epidermis: 6-0 nylon	No tape	Unspecified longitudinal tension-reducing tape	After suture removal; Not reported	Patient and observer scar assessment scale; Scar width	12 months

Legend:

*Ethicon, USA

^3M, USA

### Microporous tape

Microporous tapes were most frequently used for post-operative scar cosmesis modulation. Five studies (Table 1) with 217 patients received this intervention on the abdomen (n=3), breast (n=1) and limbs (n=1). Reported results from these studies were favourable.

Ilori et al (2022)^15^ and Atkinson et al (2005)^16^ both compared scar cosmesis in patients who received microporous taping to controls with no taping, after removal of sutures or staples that were used for primary closure. Despite different outcome measurements (Table 1), both studies reported significant improvements when using micropore tape, supporting their efficacy in scar modulation. Ilori et al (2022)^15^ reported that 97.6% of scars were identified to be of normal morphology at 6 weeks and 3 months, and this increased to 100% by 6 months post-operation. On the other hand, only 48.8% of scars that did not receive taping interventions were reported to be classified as normal at 6 weeks post-operation, with this percentage declining to 19.5% by the end of 6 months. Similarly significant improvements were also reported with scar width and height when micropore tapes were applied. Atkinson et al (2005)^16^ compared the rates of scar volume decrease in patients who received microporous taping to controls with no taping. In patients who strictly followed the taping protocol, mixed model analysis indicated that microporous taping led to higher rates of scar decrease although significance was not reached (p=0.15). Interestingly, there was a significant correlation between measured scar volume and assessor scar rating (p<0.001). Moreover, the odds of developing a hypertrophic scar were 13.6 times higher in the control group (CI 3.6 – 66.9).

Chen et al (2001),^17^ Gallo et al (2025)^18^ and Widgerow et al (2009)^19^ compared microporous tapes to other techniques of closure. Chen et al (2001)^17^ found the use of microporous tapes to close wounds resulted in similar scar widths when compared to the use of silk sutures, although a higher proportion of patients reported satisfaction with the scar’s appearance when applying tapes (98% vs 92%, p=0.03).^17^ Gallo et al (2025)^18^ compared the use of closed incision negative pressure therapy versus taping as an adjunct to primary abdominal wound closure. They found that SCAR-Q appearance scores at 1 week, 3 months and 6 months post-operation were higher in the adhesive group (32.9 vs 23.5, 37.7 vs 34.6 and 35.0 vs 29.7 respectively), although statistical analysis was not conducted as this was a pilot randomized control trial.^18^ Widgerow et al (2009)^19^ compared the use of microporous tape to microporous tape and scar gel for the closure of breast incisions. The latter intervention yielded significantly superior cosmetic results at two months (p=0.032), but differences were not significant (p=0.06) at 6 months after surgery. The authors commented that the low sample size of 10 patients in this arm was due to several patients being unwilling to continue the trial, as they believed the intervention of both tape and scar gel led to such an ‘evident’ improvement.

### Steri-strips

Five studies (n=159, Table 2) reported the effect of Steri-Strips on scar appearance for the chest (n=2), abdomen (n=1), lower limb (n=1) and neck (n=1). Lazar et al (2011)^20^ and Gevel et al (2010)^21^ demonstrated no significant differences between groups that applied post-operative Steri-strips and control groups that received no taping interventions. Rhamdan et al (2013)^22^ and O’Leary et al (2013)^23^ compared scar appearance after Steri-strip use against suturing. The former reported that there was no significant difference in appearance between the two closure modalities. Furthermore, they also found no significant differences in wound complication rates, when using steri-strips to close lower limb incisions. O’Leary et al (2013)^23^ demonstrated a significant improvement with Steri-Strips when using the Likert scale to rate scar appearance (3.3 vs 3.0, p=0.046), but did not find a significant improvement when using the Hollander Cosmesis scale to evaluate scar appearance (4.6 vs 3.7, p=0.12).

Lin et al (2020)^12^ compared the efficacy of Steri-Strips against silicone sheets on scar appearance. Both adjuncts were applied 1 week post-operatively and continuously reapplied for 3 months to modulate scar formation. The Visual Analogue Scale, rated from 1–10, demonstrated significant cosmetic improvements with silicone tape at both 6 months (6.81 vs 6.19, p=0.03) and 12 months (6.88 vs 6.20, p=0.04) post-operation. The Vancouver Scar Scales yielded no statistically discernible differences when using silicone or Steri-strips.

### Other tapes

Ishii et al (2021)^24^ performed a prospective comparative cohort comparing Yu-ki ban® and Atofine™ tapes on breast surgical incisions. The latter was found to have a significantly lower incidence of contact dermatitis (6.7% vs 44.8%, p<0.05), as well as improved aesthetic results, particularly with regards to rates of skin pigmentation (6.7% vs 34.5%, p<0.05).

Klingenstein et al (2022)^25^ analysed scar cosmesis between one group which used Steri-strips and Kinesio taping compared to another group which only used Steri-strips. The addition of Kinesio taping appeared to only improve scar colour – 62.5% of scars were assessed to show erythema, in comparison to the 87.5% recorded in the control group (p=0.031). No other increases in observer rated scar scores or differences in scar length were reported between the two groups.

Su et al (2025)^26^ and Shiva et al (2023)^27^ were two exceptions where the authors did not specify which type of tape was used. The former longitudinally applied ‘tension-reducing tape’ in addition to modified buried vertical mattress sutures and saw statistically significant improvements in both Patient and Observer Scar Assessment Scales (15.35 vs 12.24, p=0.001; 14.41 vs 11.21, p=0.001), as well as scar width (2.49mm vs 1.31mm, p=0.001), as opposed to using sutures alone. Shiva et al (2023)^27^ compared the cosmetic outcomes of three groups including tape, N-butylcyanoacrylate and suturing. Although these authors reported that adhesive glue had the best outcomes according to Hollander, VAS, patient and surgeon satisfaction scores, taping interventions demonstrated superior cosmetic outcomes in comparison to suturing. By day 90, tape application resulted in a higher proportion of scars being rated as ‘optimal’ using the Hollander Wound Evaluation Score (36.4% vs 13.6%, p=0.0012). Moreover, the visual analogue score on day 90 was also significantly higher for tapes than for sutures (67.26 vs 65.28, p<0.0001).

### Risk of bias assessment

Of the 11 included studies that underwent the Cochrane Risk of Bias Tool 2.0 evaluation, the majority (n=6) were deemed to be at a high risk of bias, and the remaining five to be at a moderate risk of bias. The three non-randomised studies were assessed using the ROBINS-I tool, all of which displayed a serious risk of bias. The assessment breakdown for all included studies can be found in Supplementary Figure B.

## Discussion

### Principal findings

This systematic review synthesised evidence from 14 studies analysing the outcomes of tape application on scar cosmesis. The use of tapes led to improved or equivalent outcomes compared to controls, supporting the hypothesis that mechanical tension plays a key role in modulating scar formation. However, the included studies displayed a variety of heterogeneous factors (Table 1; Table 2; Table 3) including inconsistent follow-up timings, outcome assessment tools, anatomical regions, tape duration and heterogeneity in closure techniques. The varied outcome assessment tools in particular made quantitative pooling of results difficult, and therefore a meta-analysis was not able to be performed.

O’Reilly et al (2021)^14^ performed a similar comprehensive review investigating the efficacy of applying tapes, although fewer studies (n=9), with a lower level of evidence, were included. These findings from O’Reilly were also similarly encouraging, suggesting that tapes appear to be an effective modality for scar cosmesis modulation. Stratis et al (2024)^28^ focused exclusively on the use of paper tapes for scar modulation, and similarly supported the aforementioned findings, although a need for additional, higher evidence papers was noted.

### Tape mechanisms of action

Although the specific pathophysiology of hypertrophic and keloid scar formation remains to be fully delineated, tension in the healing wound is a known, contributing factor.^29^ During the initial stages of the healing process, type III collagen fibres are thought to be over-deposited to compensate for their structural weakness, resulting in significantly hypertrophied scars.^30^ Although these type III fibres are eventually replaced by stronger type I fibres^31^ early tension control through the application of tape is thought to result in more ordered collagen deposition^14^ and improved cosmesis. Of note, variation in closure techniques may also elicit differing degrees of tension the healing wound, thereby modulating scar cosmesis. As such, the heterogenous nature of the included closure techniques (Table 1; Table 2; Table 3) may also be modulating scar appearance to differing degrees.

Tape permeability should also be considered, as factors such as oxygen tension and humidity may influence scar appearance. Some evidence suggests that low oxygen tension in the wound may promote hypertrophic scar development,^32^ through promotion of excessive collagen deposition.^16^^,^^33^ On the other hand, increased moisture may result in the downregulation of fibroblast mediated collagen and extracellular matrix deposition, resulting in a lesser inflammatory response and a shorter proliferative phase of the scar.^34^, ^35^, ^36^ The barrier function of tape may also contribute to protecting the healing wound from external irritants that may interfere with the healing process.

The timing of tape application may also be a significant contributing factor. The remodelling phase of scars lasts up to 12 weeks postoperatively,^37^ and the length of time which the tape was applied throughout this process may lead to clinically different results. Of note, the four Steri-strip papers which found no discernible difference compared to sutures were applied only for a maximum of two weeks. However, Lin et al (2021)^38^ administered Steri-strips for three months, and reported results that were clinically similar to gold-standard silicone tapes. The remodelling phase of scars is thought to start 2-3 weeks after the initial injury, and the scar’s maximal strength is reached by 12 weeks post injury.^37^ As such, further studies investigating protocols of tape application for longer periods of time is necessary for optimal scar prophylaxis guideline recommendations.

### Tape categories

The efficacy of silicone sheets in managing hypertrophic and keloid scars is well established and is currently accepted as a first-line treatment.^5^^,^^39^ The mechanism of silicone sheet action is not fully understood, and this material presents with multiple constraints. For example, silicone sheets are costly, with reported prices up to £34.00 per sheet and regular hygienic maintenance is required for the avoidance of complications such as skin maceration and breakdown.^40^ Patients are often asked to wear them for up to 24 hours daily, for 2-3 months at a time.^41^ Perspiration, particularly during the summer months, can lead to easy dislodgement of these sheets and can also increase incidences of pruritus.^42^

There is no consensus on other types of taping interventions. From the included studies, Steri-strips and microporous tapes appear to be the most widely available alternatives to silicone sheets. Although the Steri-strips were mainly used as an alternative to suture closure, Lin et al (2020)^38^ compared these strips to silicone sheets, and found a clinically insignificant difference between them. The use of steri-strips in place of silicone sheets would also have significant cost saving benefits, as they can cost less than £1.5 for a 10-piece pack. However, additional studies replicating this comparison are needed before recommendations can be made.

Microporous tapes are similarly inexpensive tapes costing less than £0.04/metre. Although Ishii et al (2021)^24^ focused on cosmetic outcomes between Atofine™ and Yu-ki ban® tapes, favouring the former, they also carried out a tape characteristic analysis on Atofine™, Yu-ki ban®, Micropore™ and silicone sheets. Based on their findings, the authors recommended the use of a tape that possesses strong adhesive properties and low keratinocyte removal when taken off to decrease incidences of contact dermatitis and improve scar cosmesis. Previous findings support the presence of a moist healing environment, such as the currently used silicone sheets.^43^ However, Ishii et al (2021)^24^ suggests that tapes that lead to excessive moisture retention at site of healing may actually be detrimental to scar appearance and increase the risk of dermatitis. A comparable analysis of Steri-Strip characteristics would therefore be valuable, and further studies analysing tape characteristics would be useful.

### Alternatives to taping

Several limitations of using tapes need to be considered. There are difficulties with applying tape to highly mobile or non-haemostatic/weeping/oily areas that can interfere with its adhesiveness. Common complications include allergic reactions and the formation of tension blisters. Although many of the included studies report a neutral or favourable scar appearance when tape was used, long-term application in certain patients can lead to contact dermatitis and worsen the pigmentation of scars.^24^ Future evaluations with alternatives to taping are required.

Gallo et al (2025)^18^ evaluated the use of negative pressure wound therapy (NPWT) on scars, comparing outcomes to the use of microporous tapes. Although no statistical analysis was performed in this study, SCAR-Q results appeared to be higher than that achieved with the microporous taping group. NPWT provides a similar mechanical tension by facilitating the apposition of the wound site, as well as providing negative pressure to reduce oedema and promote tissue granulation. Preliminary analyses investigating NPWT and scar modulation with data from moderate-level studies showed promising results,^44^ although a recent randomised controlled trial suggested that NPWT provided little benefit for improving scar appearance.

Shiva et al (2023)^27^ reported that the use of N-butylcyanoacrylate led to superior scar cosmetic outcomes in comparison to tapes. Glue adhesives provide tensile forces around a healing wound in a manner that is similar to tapes, and are commonly used for small superficial lacerations. They also confer additional advantages such as low maintenance, possessing antimicrobial properties and easy administration.^45^ Preliminary evidence investigating its efficacy in modulating scar cosmesis is limited, although several studies generally demonstrate favourable cosmetic results that are on par with taping in children.^46^^,^^47^

### Limitations

Although the level of evidence for the included studies was generally quite high, several limitations of this systematic review must be mentioned. The majority of included studies were at a high or serious risk of bias, and findings should be interpreted cautiously. The significant heterogeneity of outcomes made synthesis, let alone inter-study comparisons not possible. There was also significant variation with regards to the location of incisions (Table 1; Table 2; Table 3), which made comparison difficult as skin properties, including thickness, vary depending on the anatomical region.^22^

Details were lacking with regards to incision length and the surgical closure methods were inconsistent or not specified. Wounds should be sutured in layers unless they are very small. Variations in closure methods will undoubtedly contribute to different levels of tension applied at primarily closed wounds, leading to changes of scar appearance that are independent of the tape used, thus making meaningful comparisons difficult. Moreover, known risk factors such as smoking, diabetes and ethnicity that can contribute to adverse scar formation were poorly reported in the majority of studies. The inconsistent recording of these known contributors needs to be considered when interpreting these results.

## Conclusion

Tapes appear to demonstrate encouraging results when used as a modality for modulating scar cosmesis following primary wound closure. All included studies reported either improved or equivalent cosmetic outcomes when compared to controls, suggesting that tension may play an important role in controlling scar formation. However, other factors may still play a role. Further randomised controlled trials studies using a consistent primary closure method in layers and directly comparing different types of tapes including silicone sheets using longer follow up and standardised reporting outcomes are needed.

## Financial disclosure statement

The authors have nothing to disclose.

## Presentations

Not presented at the time of submission.

## Funding

None.

## Ethical approval

Not required.

## Declaration of competing interest

None declared.

## Appendix Supplementary materials

Image, application 1

Image, application 2
