# Supplementary material for: Cosmetic outcomes of postoperative scar taping after surgical incisions: A systematic review
Source: JPRAS Open. 2026 Jul 8;51:374–86. doi: 10.1016/j.jpra.2026.07.002 (PMC13445350; doi:10.1016/j.jpra.2026.07.002)
Supplement: Supplementary file 1 [file mmc1.docx]

**Supplementary Figure A**

EMBASE:

| 1 | scar/ |
| --- | --- |
| 2 | burn scar/ or burn surgery/ or burn/ |
| 3 | surgical wound/ or wound healing/ |
| 4 | cicatri*.mp. |
| 5 | scar*.mp. |
| 6 | hypertroph*.mp. |
| 7 | burn*.mp. |
| 8 | surgical wound*.mp. |
| 9 | surgical incision*.mp. |
| 10 | 1 or 2 or 3 or 4 or 5 or 6 or 7 or 8 or 9 |
| 11 | surgical tape/ |
| 12 | athletic tape/ |
| 13 | tap*.mp. |
| 14 | MeFix.mp. |
| 15 | Fixomull.mp. |
| 16 | Hypafix.mp. |
| 17 | 11 or 12 or 13 or 14 or 15 or 16 |
| 18 | 10 and 17 |

MEDLINE:

| 1 | Cicatrix/ |
| --- | --- |
| 2 | Burns/ |
| 3 | Wound Healing/ or Surgical Wound/ |
| 4 | cicatri*.mp. |
| 5 | scar*.mp. |
| 6 | hypertroph*.mp. or Hypertrophy/ |
| 7 | burn*.mp. |
| 8 | surgical wound*.mp. |
| 9 | surgical incision*.mp. |
| 10 | 1 or 2 or 3 or 4 or 5 or 6 or 7 or 8 or 9 |
| 11 | Surgical Tape/ |
| 12 | Athletic Tape/ |
| 13 | tap*.mp. |
| 14 | mefix.mp. |
| 15 | fixomull.mp. |
| 16 | hypafix.mp. |
| 17 | 11 or 12 or 13 or 14 or 15 or 16 |
| 18 | 10 and 17 |

Cochrane:

#1	MeSH descriptor: [Cicatrix] explode all trees

#2	MeSH descriptor: [Burns] explode all trees

#3	MeSH descriptor: [Surgical Wound] explode all trees

#4	cicatri*

#5	scar*

#6	hypertroph*

#7	burn*

#8	surgical wound*

#9	surgical incision*

#10	#1 OR #2 OR #3 OR #4 OR #5 OR #6 OR #7 OR #8 OR #9

#11	MeSH descriptor: [Surgical Tape] explode all trees

#12	MeSH descriptor: [Athletic Tape] explode all trees

#13	tap*

#14	MeFix

#15	Fixomull

#16	Hypafix

#17	#11 OR #12 OR #13 OR #14 OR #15 OR #16

#18	#10 AND #17
