# Supplementary material for: Cosmetic outcomes of postoperative scar taping after surgical incisions: A systematic review
Source: JPRAS Open. 2026 Jul 8;51:374–86. doi: 10.1016/j.jpra.2026.07.002 (PMC13445350; doi:10.1016/j.jpra.2026.07.002)
Supplement: Supplementary file 2 [file mmc2.docx]

**Supplementary Figure B**

Cochrane Risk of Bias 2.0 Results:

ROBINS-I Tool Results:
